# Maternal depressive symptoms are negatively associated with child growth and development: Evidence from rural India

**DOI:** 10.1111/mcn.12621

**Published:** 2018-05-17

**Authors:** Phuong Hong Nguyen, Jed Friedman, Mohini Kak, Purnima Menon, Harold Alderman

**Affiliations:** ^1^ International Food Policy Research Institute Washington DC USA; ^2^ The World Bank Washington DC USA

**Keywords:** child development, child undernutrition, depression, India, stunting

## Abstract

Maternal depression has been suggested as a risk factor for both poor child growth and development in many low‐ and middle‐income countries, but the validity of many studies is hindered by small sample sizes, varying cut‐offs used in depression diagnostics, and incomplete control of confounding factors. This study examines the association between maternal depressive symptoms (MDSs) and child physical growth and cognitive development in Madhya Pradesh, India, where poverty, malnutrition, and poor mental health coexist. Data were from a baseline household survey (*n* = 2,934) of a randomized controlled trial assessing an early childhood development programme. Multivariate linear and logistic regression analyses were conducted, adjusting for socio‐economic factors to avoid confounding the association of mental health and child outcomes. MDS (measured using the Center for Epidemiologic Studies Short Depression Scale) was categorized as low, medium, and high in 47%, 42%, and 10% of mothers, respectively. The prevalence of child developmental delay ranged from 16% to 27% for various development domains. Compared with children of mothers with low MDS, those of high MDS mothers had lower height‐for‐age, weight‐for‐age, and weight‐for‐height *z*‐scores (0.22, 0.21, and 0.15, respectively), a higher rate of stunting and underweight (~1.5 times), and higher rate of developmental delay (partial adjusted odds ratio ranged from 1.3–1.8 for different development domains and fully adjusted odds ratio = 1.4 for fine motor). Our results—that MDS is significantly associated with both child undernutrition and development delay—add to the call for practical interventions to address maternal depression to simultaneously address multiple outcomes for both women and children.

**Key messages**

- Maternal depressive symptoms are significantly associated with child undernutrition in India, with possible mechanisms operating though poorer home environment, less engagement with children, lower use of health services, and suboptimal complementary feeding practices.
- Depressive symptoms also are associated with development delay among children <4 years, although the strength of the association depends on the domain of development considered.
- As poverty, poor mental health, and undernutrition coexist in India, it is crucial to consider maternal depression in endeavours to simultaneously address multiple outcomes for both women and children.

## 1 INTRODUCTION

Maternal depression is a risk factor for undernutrition as well as for delayed cognitive development in many low‐ and middle‐income countries (Britto et al., 2017; Surkan, Kennedy, Hurley, & Black, 2011). Both antenatal and post‐natal depression can influence a child, albeit through different pathways (Soe et al., 2016). Maternal depression may affect child outcomes from very early during pregnancy (through altered placental function, epigenetic changes, and stress reactivity) to post‐natal period, infancy, and childhood (via altered mother–child interactions, less affection and responsiveness, poor psychosocial stimulation, inadequate feeding, poor hygiene, and health‐seeking practices; Herba, Glover, Ramchandani, & Rondon, 2016). Given that the weighted mean prevalence of antenatal and post‐natal depression in low‐ and middle‐income countries has been estimated at 25.3% and 19.6%, respectively (Gelaye, Rondon, Araya, & Williams, 2016), with prevalence of depressive symptoms in South Asian countries often even higher (Parsons, Young, Rochat, Kringelbach, & Stein, 2012), an understanding of these risks can contribute to understanding the barriers to improve nutrition and child development in countries where undernutrition remains a stubborn problem.

The apparently clear observational association of maternal depressive symptoms and child stature as indicated by an odds ratio (OR) for stunting of 1.4 in a meta‐analysis belies a fair amount of variation across settings (Surkan et al., 2011). For example, only five of the 12 studies of stunting employed in that meta‐analysis found a statistically significant relationship with maternal depression. Dissimilarities in results across countries reflect, in part, differences in inclusion of covariates and in the cut‐offs used in the various diagnostic and self‐reported measures of depression as well as intrinsic differences across sample populations. Regarding child development, the relatively few studies in low‐ and middle‐income contexts also suggest an association between maternal depression and child cognitive development, but validity of these studies is hindered by small sample sizes and incomplete control of confounding factors (Gelaye et al., 2016). Further results from disparate settings and with meaningful covariate controls are necessary to identify the conditions under which maternal depression most adversely impacts child growth and development.

This study examines the association between maternal depressive symptoms and various measures of child growth and development in a large sample drawn from an understudied population—rural India—specifically two rural districts in the central Indian state of Madhya Pradesh. Relatively few studies that investigate this question are able to explore in a flexible manner the conditional association between maternal depressive symptoms and measures of both physical growth and cognitive development. Further, as poverty, deprivation, and poor mental health are often coincident (Das, Do, Friedman, Mckenzie, & Scott, 2007; Lund et al., 2010), a robust set of socio‐economic controls are included to avoid confounding the association of mental health and child outcomes by socio‐economic factors that may simultaneously determine both.

## 2 DATA AND METHODS

### 2.1 Data source and study population

The data were collected in the baseline household survey of a clustered randomized controlled trial. This study assesses a pilot programme implemented in Madhya Pradesh, India, which aims to improve early childhood development outcomes with the introduction of day care delivered by the Integrated Child Development Scheme at Anganwadi centres (AWCs) in addition to the supplementary nutrition and health services already being provided. The household survey was conducted in Dhar and Singrauli districts in 2014. 1The trial, Making Integration the Operative Concept in the Indian Integrated Child Development Services, is AEARCTR‐0000967 registered at the AEA RCT Registry.


The sample of intervention and control AWCs in these districts was selected based on two‐stage randomization of the first treatment and control sectors followed by a random selection of three to four centres within each study sector, resulting in a well‐balanced sample between treatment and control centres with respect to catchment size and prevalence of underweight children. 2Districts are administratively subdivided into blocks, and blocks are further subdivided into sectors. Singrauli has 53 sectors, and Dhar has 131. They both average a bit more than 28 AWCs per sector. The survey first involved the complete listing of AWC catchment areas (200 enumeration areas) for eligible households (those currently with a child or children between the ages of 6 and 59 months). This population constituted the sampling frame for the household interview. Fifteen households in each AWC catchment area were randomly selected from this list. As a few catchment areas had less than 15 eligible households, 2,934 mother–child pairs participated in the survey. Mothers were informed about the purpose of the study, and oral informed consent was obtained from all participants. Data were collected via face‐to‐face interviews using a structured questionnaire. The survey received ethical approval from the Institutional Review Board from the International Food Policy Research Institute in the United States and the Centre for Media Studies in New Delhi, India.

### 2.2 Child anthropometry

We examine the associations of maternal depressive symptoms (MDSs) with child growth, using both continuous *z*‐scores based on the 2006 World Health Organization (WHO) child growth standards (de Onis et al., 2009) as well as measures of undernutrition for individuals more than two standard deviations below the medians of these standards.

Child anthropometry was obtained by trained field staff using standard methods (Cogill, 2003). As the trial assessed the impact of day care centres, the survey teams were instructed to select an index child from each household in the age range of 6–48 months. If there were more than one child in this age range, the older child was considered the index child. This choice was to facilitate comparisons among children who had the opportunity to participate in day care centres—a focus of the study on integrated child development services. Children's weight was measured by Tanita weighing scales, accurate to 100 g. For height, supine length was measured for children from 0 to 23.9 months and standing height was measured for children ≥24 months old, using standardized length boards, which were precise to 1 mm. Children's weight and length/height measurements were then converted into height‐for‐age *z*‐score (HAZ), weight‐for‐age *z*‐score (WAZ), and weight‐for‐length/height *z*‐score (WHZ), according to WHO (2010) child growth standards. Stunting was defined as HAZ below −2 *z*‐score, underweight as WAZ < −2, and wasting as WHZ < −2 (WHO, 2010).

### 2.3 Child development

Child development was measured by a parent‐reported instrument, the Ages and Stages Questionnaire, Third Edition (ASQ‐3), which includes five domains, namely, communication, gross motor, fine motor, problem solving, and personal social skills (Squires & Bricker, 2009). The ASQ‐3 has been standardized on children aged from 1 month to 5.5 years. Each questionnaire contains 30 items, grouped by developmental domains (each domain has six items), about a child's everyday activities. Mothers were asked to select the most appropriate response (“yes,” “sometime,” or “not yet”) based on the previous observations of the child's ability or inability to perform daily activities expected of the specific age group. The total age‐standardized score for each domain is 60. These scores were compared with the cut‐off for the child‐specific age group to identify children with developmental delay that need further assessment. The ASQ‐3 has been widely used in many population and has been approved as a valid and reliable tool to screen child developmental delay (Fernald, Kariger, Engle, & Raikes, 2009; Kvestad et al., 2013). It has also been used as an outcome indicator of adverse effects of maternal depressive symptoms (Junge et al., 2017).

### 2.4 Maternal symptoms of depression

MDSs were measured using the Center for Epidemiologic Studies Short Depression Scale (CES‐D‐10), an adaptation of the 20‐question scale in common use (Radloff, 1977). The shortened set of questions has been validated in various settings (Irwin, Artin, & Oxman, 1999; Zhang et al., 2012), including India (Kumar, Nakulan, Thoppil, Parassery, & Kunnukattil, 2016). The CES‐D‐10 scale includes a 10‐item checklist that measures symptoms of depression experienced over the past week, including feelings of bother, distraction, hopelessness, restlessness, and sleep disturbance. Each item can be scored on a scale of 0–3, yielding a range for the possible summary score from 0 to 30, with higher scores indicating increased severity of depressive symptoms. Some studies reduce 0–3 responses to yes/no dichotomies yielding a possible total score of 0–10 (Irwin et al., 1999; Kumar et al., 2016). We conduct analysis on the full score, but all reported results are robust to such a score truncation.

The CES‐D‐10 scale was translated into Hindi and checked for accuracy through back translation and pilot testing. Interviewers were trained on the protection of interviewee privacy and data confidentiality.

### 2.5 Covariates

Household characteristics examined as control variables included household composition (household size, number of children under 5 years, number of working members aged 15–64 years old, and female headed household), socio‐economic status (SES), and distance from household to the nearest AWC. The SES index was constructed using principal components analysis of variables such as ownership of a house and land, housing quality (construction materials for floor, roof, and wall), access to services (water, electricity, and sanitation services), and household assets (different types of durable goods and animals; Filmer & Pritchett, 2001; Vyas & Kumaranayake, 2006). The first factor derived from component scores was used to classify household SES into quartiles.

Maternal characteristics included age, education (categorized as no schooling, primary school—5 years of schooling, middle school—6–9 years, and high school or higher—≥10 years), and social group (tribal or other). At the child level, we adjusted for child age and sex in the analyses.

We also measure activities that may be influenced by MDS, including home environment, mother–child engagement, child feeding, and use of health services. Home environment was assessed by the presence of learning materials (number of picture books) and toys (homemade toys, manufactured toys, and household objects) and whether the child was left alone with siblings. Mother–child engagement was measured by items related to whether a mother interacted with her child in various activities (did she read books, tell stories, sing songs, play with child, name things/counted, or draw with the child) in the past 3 days. Child feeding was assessed by the number of food groups children consumed in the past 24 hours based WHO (2008) recommendations. Use of health services was measured by whether women received counselling on child health and nutrition at the AWC.

### 2.6 Statistical analysis

The association of MDS with child growth and child development and also with maternal and household characteristics, home environment, mother–child engagement, child feeding, and use of health services was indicated using *t* tests of differences in means by MDS categories for continuous variables and chi‐square tests for categorical variables. Multivariate regression models were used to examine the relationship of MDS with child growth and child development, using these outcomes both as continuous scores as well as in terms of the presence or absence of undernutrition (<−2 *SD*s) or of developmental delays, adjusting for several potential confounding factors at household, maternal, and child levels as mentioned above and accounting for centres as a random effect with a cluster sandwich estimator clustered at the AWC catchment level.

Many of the previous studies collapse the CES‐D‐10 scores into a binary indicator with a local cut‐off that represents a likely diagnosis of depression (Irwin et al., 1999; Kumar et al., 2016). However, for our purpose of understanding depressive symptoms as a risk factor, we instead examined the association between MDS and child development in a flexible manner. We first investigated the relation between MDS and child outcomes in a nonparametric manner then divided the distribution of observed CES‐D raw scores into deciles (Tables S1 and S2). We observed that only mothers in the highest decile of MDS had children with growth deficits that were greater than in other MDS groups. This suggested a relationship between maternal depression and physical growth of children among the mothers who report only the most severe depressive symptoms. In contrast, for child development, the lower score (capturing developmental delay) emerged with mothers at a medium level of MDS. Therefore, we decided to adopt a flexible specification with three cut‐offs to capture this heterogeneity in the relation between MDS and child development: low (first five deciles, score ≤ 8), medium (next four deciles, score 9–14), and high (highest decile, score ≥ 15). We also conducted sensitivity analysis, applying the same method to collapse the CES‐D‐10 scores into a binary indicator with a local cut‐off of ≥4 (Kumar et al., 2016), which is roughly at the lowest quartile in our sample. This cut‐off includes mild, moderate, and severe depression under other criteria for diagnosis reported in that study, although cut‐offs for moderate and severe depression were not reported. All statistical analyses were carried out using Stata version 14 software.

## 3 RESULTS

### 3.1 Sample characteristics

Characteristics of the study population, overall and by the three MDS categories, are presented in Table 1. The average household size was 7, with 1.6 children <5 years and 3.7 members in working age range. Very few households had the mother as household head (<5%). The mean age of mothers at the time of the survey was approximately 26 years. Overall, 47% of mothers had no schooling and only 11% completed high school or higher education. More than a half of the study population belonged to a tribal community. The average child age was 30 months with roughly equal numbers of male and female children.

**Table 1:** Sample characteristics, total and by maternal depressive symptoms

	Total	Low depressive scores (≤8)	Medium depressive scores (9–14)	High depressive scores (≥15)
	(*n* = 2,930)	(*n* = 1379, 47.1%)	(*n* = 1238, 42.3%)	(*n* = 313, 10.7%)
Household factors				
Household size	6.77 ± 2.89	6.80 ± 3.08	6.79 ± 2.69	6.58 ± 2.75
Number of children <5 years	1.60 ± 0.73	1.60 ± 0.75	1.59 ± 0.71	1.60 ± 0.70
Number of adult caregiver (>18 years)	3.67 ± 1.92	3.76 ± 2.02	3.67 ± 1.83	3.33 ± 1.72**
Female headed household	3.86	4.86	2.67	4.15*
Social economic status				
Low	25.09	21.90	27.71	28.75
Medium	25.02	21.75	26.58	33.23
High	24.95	24.87	24.56	26.84
Highest	24.95	31.47	21.16	11.18***
Distance from house to AWC	5.93 ± 25.05	4.52 ± 20.15	7.21 ± 29.46	7.10 ± 25.46*
Maternal factors				
Maternal age (years)	26.22 ± 5.36	25.80 ± 5.06	26.42 ± 5.62	27.22 ± 5.37***
Maternal education				
No schooling	47.00	38.50	52.52	62.70
1–5 years	21.44	21.98	21.54	18.65
6–9 years	20.24	23.44	17.89	15.43
≥10 years	11.32	16.08	8.05	3.22***
Maternal occupation				
Housewife	53.24	60.63	47.40	43.73
Farmer	22.47	19.14	24.47	29.26***
Others	24.29	20.23	28.13	27.01***
Social groups				
Tribal	54.95	48.66	59.77	63.58***
Other	45.05	51.34	40.23	36.42***
Child factors				
Child age (months)	30.47 ± 11.21	29.96 ± 11.41	30.99 ± 11.04	30.61 ± 10.89
Child sex (male)	49.73	48.44	50.48	52.40
Age of youngest index child	21.42 ± 12.82	21.13 ± 12.68	21.70 ± 13.05	21.54 ± 12.55
Home environment (score 1–5)	2.89 ± 0.96	2.89 ± 0.96	2.73 ± 0.99	2.36 ± 0.83***
Mother engagement (score 1–5)	1.02 ± 1.25	1.02 ± 1.25	1.01 ± 1.11	0.71 ± 0.95***
Child feeding practices (no. of food groups consumed)	3.26 ± 1.16	3.26 ± 1.16	3.13 ± 1.25	3.07 ± 1.18**
Received counselling at the AWC	47.50	47.50	50.73	38.02***

*Note*. *p* values derive from *F* tests testing the joint significance of the characteristics in relation to the maternal depressive symptoms. AWC = Anganwadi centre.

* *p* < .05.

** *p* < .01.

*** *p* < .001.

The average MDS score is 9.2 (range 1–24), with 47% was categorized as low MDS, 42% as medium MDS, and 10% as high MDS. Women with high MDS were on average, were older, had lower education levels, and were more likely to be working as farmers and from a tribal community. They also lived in households with lower SES, fewer adults, and resided a further distance from the AWC (as the AWC is generally centrally located, this is taken as measure of geographic remoteness). Furthermore, mothers with high MDS reported a poorer home environment, less engagement with children, and lower use of health services, compared with low MDS mothers. Children of mothers with high MDS also had poorer dietary diversity.

### 3.2 Associations between maternal depressive symptoms and child growth

**Figure 1:**
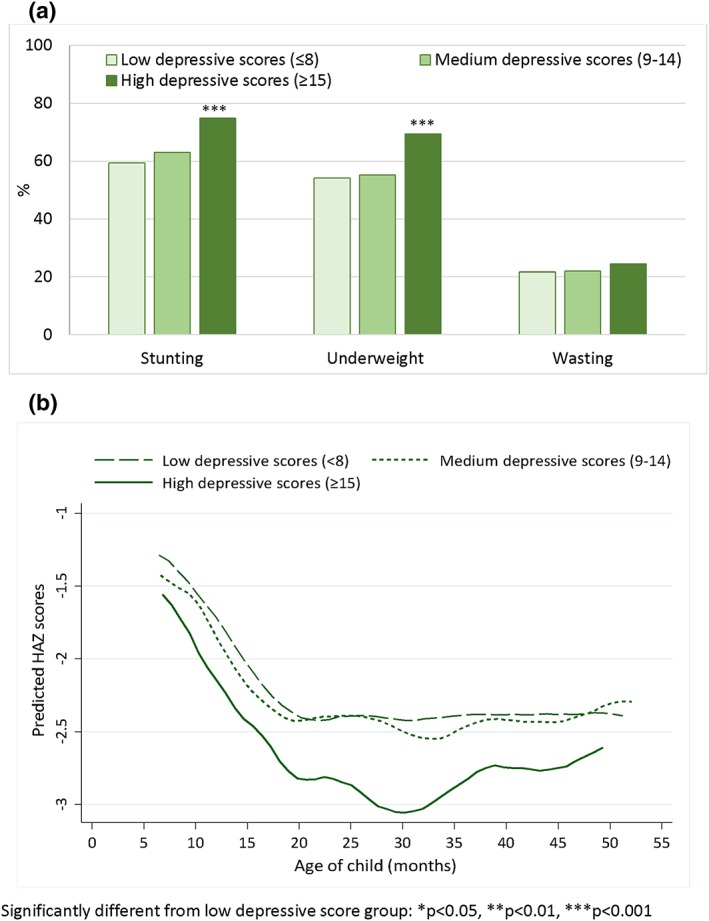
Association between maternal depressive symptoms and child growth. (a) Maternal depressive symptoms and child undernutrition. (b) Maternal depressive symptoms and child height‐for‐age *z*‐score (HAZ), by child age (in months)

Levels of undernutrition in the study sample are high, with a rate of 62.5% stunting, 56.3% underweight, and 22.2% wasting. However, given the focus on index children, the sample is not distributed evenly across age among children 0–5 years. Because, in general, stunting increases as a child ages in the first 1,000 days, stunting rates are expected to be high compared with conventional reporting such as commonly presented from Demographic and Health Surveys. Children of mothers with high MDS had a higher rate of stunting and underweight (Figure 1a) than their counterparts, with the odds ~2 times in models adjusted for child age and sex, and ~1.4 to 1.5 times in fully adjusted models (Table 2). There was no significant association between maternal MDS and wasting.

**Table 2:** Multivariate logit regression checking association between maternal depression and child undernutrition

	Stunting		Underweight		Wasting	
	Model 1	Model 2	Model 1	Model 2	Model 1	Model 2
	OR [95% CI]	OR [95% CI]	OR [95% CI]	OR [95% CI]	OR [95% CI]	OR [95% CI]
Maternal depressive scores						
Low depressive scores (≤8)	Ref.	Ref.	Ref.	Ref.	Ref.	Ref.
Medium depressive scores (9–14)	1.15 [0.95, 1.39]	0.94 [0.78, 1.14]	1.04 [0.88, 1.23]	0.83* [0.70, 0.99]	1.04 [0.86, 1.26]	0.91 [0.75, 1.11]
High depressive scores (≥15)	2.05*** [1.53, 2.76]	1.47* [1.09, 1.98]	1.93*** [1.49, 2.49]	1.39* [1.08, 1.80]	1.19 [0.85, 1.66]	0.99 [0.72, 1.37]

*Note*. Model 1 adjusts only for child age and gender. Model 2 adjusts for child age, gender, tribal caste, mother as household head, number of children <5, number of working age members, mother's age, mother's education, and household socio‐economic status. All models adjust for observational dependency across observations at the Anganwadi centre catchment level.

* *p* < .05.

** *p* < .01.

*** *p* < .001.

Figure 1b depicts the age profile of HAZ by the MDS category or the mother. Children in the study sample were born with low HAZ (~ −1.5), WAZ (−1.5 to −2), and WHZ (−1 to −1.5). There were rapid declines in HAZ from approximately 6 to 20 months of age, followed by growth stabilization. However, at any age, children of mothers with high MDS have the greatest growth deficits. Compared with children of mothers with low MDS, children of mothers with high MDS had lower HAZ (−0.45 *z*‐score), WAZ (−0.43), and WHZ (−0.26; Table 3). These associations were attenuated about a half after controlling for other covariates, but they remained statistical significant at −0.22 for HAZ, −0.21 for WAZ, and −0.15 for WHZ (Table 3).

**Table 3:** Multivariate linear regression for the association between maternal depressive symptoms and child anthropometry scores

	HAZ		WAZ		WHZ	
	Model 1	Model 2	Model 1	Model 2	Model 1	Model 2
	β [95% CI]	β [95% CI]	β [95% CI]	β [95% CI]	β [95% CI]	β [95% CI]
Maternal depressive scores						
Low depressive scores (≤8)	Ref.	Ref.	Ref.	Ref.	Ref.	Ref.
Medium depressive scores (9–14)	−0.07 [−0.19, 0.05]	0.06 [−0.05, 0.17]	−0.08 [−0.18, 0.03]	0.06 [−0.04, 0.15]	−0.04 [−0.13, 0.06]	0.04 [−0.05, 0.14]
High depressive scores (≥15)	−0.45*** [−0.62, −0.28]	−0.22** [−0.38, −0.06]	−0.43*** [−0.57, −0.29]	−0.21** [−0.34, −0.09]	−0.26***	−0.15*
					[−0.40, −0.11]	[−0.29, −0.01]

*Note*. Model 1 adjusts only for child age and gender. Model 2 adjusts for child age, gender, tribal caste, mother as household head, number of children <5, number of working age members, mother's age, mother's education, and household socio‐economic status. All models adjust for observational dependency across observations at the Anganwadi centre catchment level. HAZ = height‐for‐age *z*‐score; WAZ = weight‐for‐age *z*‐score; WHZ = weight‐for‐length/height *z*‐score.

* *p* < .05.

** *p* < .01.

*** *p* < .001.

### 3.3 Associations between maternal depressive symptoms and child development

**Figure 2:**
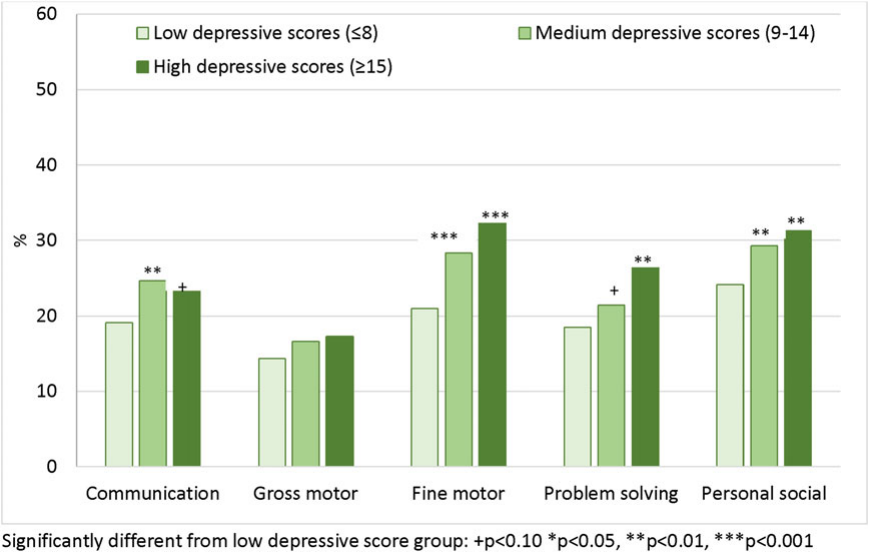
Association between maternal depressive symptoms and child development

The prevalence of child developmental delays as indicated in the ASQ assessment was 22% for communication, 16% for gross motor, 25% for fine motor, 21% for problem solving skills, and 27% for social skills. Mothers with medium and high levels of MDS had a significantly higher proportion of having a developmental delay child compared with mothers with low levels of MDS (Figure 2), with the partial adjusted OR ranged from 1.3 to 1.8 for different development domains and fully adjusted OR = 1.3 to 1.4 for fine motor (Table 4). Compared with children with low MDS mothers, those with mothers that had medium and high MDS had lower developmental score in communication, gross and fine motors, problem solving, and personal social skills (Table 5). These associations attenuated in the full model after adjusting for child, maternal, and household factors and only remained statistically significant for fine motor development (β = −1.8 for low MDS and −3.9 for high MDS). Child development results are generally robust to an analysis that discretizes the CES‐D individual questions and then adopts a cut‐off of 4 as used in a previous study (Kumar et al., 2016). However, this treatment of the data fails to capture the negative relation between MDS and child growth observed at the highest decile of depressive symptoms (Tables S3 and S4).

**Table 4:** Multivariate logit regression model for association between maternal depression and child developmental delay

	Communication		Gross motor		Fine motor		Problem solving		Personal social	
	Model 1	Model 2	Model 1	Model 2	Model 1	Model 2	Model 1	Model 2	Model 1	Model 2
	OR [95% CI]	OR [95% CI]	OR [95% CI]	OR [95% CI]	OR [95% CI]	OR [95% CI]	OR [95% CI]	OR [95% CI]	OR [95% CI]	OR [95% CI]
Maternal depressive scores										
Low depressive scores (≤8)	Ref.	Ref.	Ref.	Ref.	Ref.	Ref.	Ref.	Ref.	Ref.	Ref.
Medium depressive scores (9–14)	1.36** [1.10, 1.68]	1.18 [0.95, 1.48]	1.24† [0.99, 1.54]	1.08 [0.86, 1.36]	1.49*** [1.20, 1.85]	1.29* [1.04, 1.60]	1.19 [0.96, 1.48]	1 [0.82, 1.23]	1.29** [1.08, 1.55]	1.15 [0.96, 1.38]
High depressive scores (≥15)	1.28 [0.92, 1.77]	0.99 [0.72, 1.37]	1.28 [0.92, 1.79]	1.02 [0.72, 1.45]	1.82*** [1.36, 2.45]	1.42* [1.05, 1.91]	1.59** [1.16, 2.17]	1.22 [0.90, 1.65]	1.43* [1.04, 1.96]	1.15 [0.84, 1.59]

*Note*. Model 1 adjusts only for child age and gender. Model 2 adjusts for child age, gender, tribal caste, mother as household head, number of children <5, number of working age members, mother's age, mother's education, and household socio‐economic status. All models adjust for observational dependency across observations at the Anganwadi centre catchment level.

† *p* < .10.

* *p* < .05.

** *p* < .01.

*** *p* < .001.

**Table 5:** Multivariate linear regression model for association between maternal depressive scores and child developmental scores

	Communication		Gross motor		Fine motor		Problem solving		Personal social	
	Model 1	Model 2	Model 1	Model 2	Model 1	Model 2	Model 1	Model 2	Model 1	Model 2
	β [95% CI]	β [95% CI]	β [95% CI]	β [95% CI]	β [95% CI]	β [95% CI]	β [95% CI]	β [95% CI]	β [95% CI]	β [95% CI]
Maternal depressive scores										
Low depressive scores (≤8)	Ref.	Ref.	Ref.	Ref.	Ref.	Ref.	Ref.	Ref.	Ref.	Ref.
Medium depressive scores (9–14)	−1.92* [−3.53, −0.32]	−0.53 [−2.03, 0.97]	−1.25* [−2.48, −0.02]	−0.26 [−1.46, 0.94]	−3.51*** [−5.19, −1.82]	−1.77* [−3.23, −0.32]	−2.09** [−3.44, −0.74]	−0.62 [−1.78, 0.54]	−1.88** [−3.24, −0.52]	−0.83 [−2.07, 0.42]
High depressive scores (≥15)	−0.69 [−3.27, 1.90]	1.68 [−0.80, 4.17]	−1.47 [−3.47, 0.53]	0.38 [−1.69, 2.46]	−6.95*** [−9.51, −4.39]	−3.88** [−6.27, −1.49]	−3.73*** [−5.88, −1.57]	−1.17 [−3.14, 0.80]	−2.80* [−5.12, −0.47]	−0.93 [−3.15, 1.29]

*Note*. Model 1 adjusts only for child age and gender. Model 2 adjusts for child age, gender, tribal caste, mother as household head, number of children <5, number of working age members, mother's age, mother's education, and household socio‐economic status. All models adjust for observational dependency across observations at the Anganwadi centre catchment level.

* *p* < .05.

** *p* < .01.

*** *p* < .001.

## 4 DISCUSSION

This paper indicates a considerable public health problem of child undernutrition and delays in other dimensions of development in rural Madhya Pradesh in India. It corroborates previous findings that MDS is significantly associated with both child growth and development. Children of mothers with high MDS were approximately 2 times more likely to be stunted and underweight and 1.3–1.8 times more likely to have developmental delay in the models adjusting for child age and sex only. Although the inclusion of socio‐economic controls roughly halves the estimated association between maternal mental health and child outcomes, significant associations with physical growth and fine motor skills persist; the OR for stunting in the adjusted model is in keeping with the existing literature (Surkan et al., 2011). We also observe that physical or cognitive developmental deficits are associated with different threshold levels of depressive symptoms. For child development, the lower score for developmental delay was observed among mothers at a medium level of MDS. In contrast, poorer child growth was only observed among the mothers with the highest decile of MDS.

Our findings on the association between high MDS and child undernutrition are also consistent with previous studies in South Asia such as India (Anoop, Saravanan, Joseph, Cherian, & Jacob, 2004; Harpham, Huttly, De Silva, & Abramsky, 2005; Patel, Desouza, & Rodrigues, 2003), Bangladesh (Black, Baqui, Zaman, El Arifeen, & Black, 2009; Nguyen et al., 2014), and Pakistan (Rahman, Iqbal, Bunn, Lovel, & Harrington, 2004a; Rahman, Lovel, Bunn, Iqbal, & Harrington, 2004b). In contrast to the extensive literature on child physical growth, fewer studies have assessed child developmental outcomes in relation to maternal depression in low‐ and middle‐income countries (Gelaye et al., 2016). To our knowledge, there are only two studies in India that have been reported. One documented the significant association between post‐partum depression with reduced mental development quotient scores of infants less than 6 months (Patel et al., 2003). The second recent study reported associations between maternal mental health in the first year of life with both growth and development at age one, and this association persisted until age 5 and 8 years (Bennett, Schott, Krutikova, & Behrman, 2016).

The mechanisms by which maternal depression may create a risk of poor child outcomes are complex. Maternal care behaviours and practices are important elements for translating resources for care (including food, health care, psychosocial stimulation, and emotional support) into child well‐being (Engle, Menon, & Haddad, 1999). Maternal depression reduces a mother's ability to take adequate care of her child, which in turn can have negative effects on the child's growth and development (Rahman, Patel, Maselko, & Kirkwood, 2008; Stewart, 2007; Walker et al., 2007). The roles of care behaviours in constrained conditions such as poverty are even more important, because good maternal care can optimize the available resources to promote children's well‐being. Recent review papers have examined more comprehensive mechanisms underlying associations between maternal depression and adverse child outcomes, including both biological and psychosocial potential mechanisms from fetal programming, genetic influences, and gene–environment interactions (Herba et al., 2016), medical complications of pregnancy, the delivery of low birthweight and/or preterm infants, and environmental and nutritional influences (Gelaye et al., 2016). Our study also showed significant association between high MDS with poorer home environment, less engagement with children, lower use of health services, and suboptimal complementary feeding practices as shown by poorer dietary diversity.

The correlates of high MDS identified in this study were in line with those found in the literature. For example, a systematic review of 115 studies in low‐ and middle‐income countries noted that more than three quarters of these studies reported positive associations between a range of poverty indicators (such as education, food insecurity, housing, social class, SES, and financial stress) and poor maternal mental health (Lund et al., 2010). Poverty and low education are also well‐known as risk factors for child undernutrition and developmental delay (Black et al., 2013). The factors that we identified as correlates of high MDS in our analysis—higher maternal age, lower education levels, working as farmers, from a tribal community, lower SES, fewer adults, and further from public services such as the AWC—are predominantly nonmodifiable determinants in the short term. However, factors such as SES and proximity to public services are amenable to modification via policy instruments such as cash transfers (to improve economic status) and location of public services or improving transport conditions (to improve proximity to public services). It is also clear that some of the determinants—that is, fewer adults in the household—reflect potentially limited social support mechanisms for child care and other household or economic tasks. Although these are nonmodifiable for individual households, community‐based interventions such as support groups or child care services can help to strengthen overall support available for poor families struggling with multiple constraints.

A few limitations of this study merit further discussion. The study is cross‐sectional, and while proving evidence of an association of risk factor for child development, it is not designed to test direct causality. There is a possibility that poor developmental outcomes and maternal depression are both influenced by additional factors not included in the multivariate regressions. However, we believe that this is a limited possibility because our models include broad and robust controls. Similarly, it is possible that there is some reverse causality, that is, that poor child development contributed to maternal depression; our data do not permit assessment of this given the cross‐sectional nature. The ASQ has the convenience for field work at scale in that it is reported by mothers (Fernald et al., 2009); however, there is a slight limitation in that mothers with high MDS may report differently from those with low MDS. The ASQ is only one of a range of screening instruments useful in low‐income settings (Rubio‐Codina, Araujo, Attanasio, Munoz, & Grantham‐Mcgregor, 2016). Other instruments may shed additional light on the patterns in this study. In our sample, we had 74% with MDS ≥4; thus, using this cut‐off would not allow us to distinguish the relationship of between moderate or severe MDS and child growth, which was only observed at the highest decile. To overcome this challenge, we applied flexible methods including first investigating the nonparametric association between MDS and child outcomes, which motivated the modelling choice of deciles with a two‐part cut‐off. However, these cut‐offs may differ from other studies and thus hinder the comparison across studies.

## 5 CONCLUSIONS

In conclusion, our study results add to the accumulating body of evidence that maternal depression is a risk factor for impaired child growth and delayed child development. Our findings echo the call for practical interventions to mitigate depression directly (Rahman et al., 2013) in maternal and child health and nutrition programmes (Bhutta et al., 2008). It is possible that social safety net or social support programmes that address the modifiable determinants of maternal depression may play a complementary or synergistic role as well (Alderman & Fernald, 2017). If such programmes are feasible, they would be intrinsically joint social support, nutrition, and development endeavours. Moreover, unlike many programmes, they could simultaneously address the woman as an individual and not merely as a caregiver.

## CONFLICTS OF INTEREST

The authors declare that they have no conflicts of interest. The findings, interpretations, and conclusions expressed in this paper are entirely those of the authors. They do not necessarily represent the views of the International Bank for Reconstruction and Development/World Bank and its affiliated organizations, or those of the Executive Directors of the World Bank or the governments they represent.

## CONTRIBUTIONS

PHN contributed to developing the research questions, performed statistical analyses, interpreted the results and wrote the manuscript. JF and HA are principal investigators of the study. They contributed to developing the research questions and study design, writing the research proposal and obtaining funding, overseeing implementation of the trial, interpretation of findings, and reviewing manuscript. MK provided support for the fieldwork and administrative support and reviewed the manuscript. PM contributed to interpretation of findings and reviewed and edited the manuscript. All authors contributed to the development, review, and approval of the final manuscript.

## Supporting information

Table S1: Multivariate linear regression model for association between maternal depressive scores (by decile) and child growth
Table S2: Multivariate linear regression model for association between maternal depressive scores (by decile) and child development
Table S3: Multivariate logit regression for association between maternal depression and child undernutrition
Table S4: Multivariate logit regression model for association between maternal depression and child development
