# Supplementary material for: Maternal depressive symptoms are negatively associated with child growth and development: Evidence from rural India
Source: Matern Child Nutr. 2018 May 17;14(4):e12621. doi: 10.1111/mcn.12621 (PMC6175434; doi:10.1111/mcn.12621)
Supplement: Supplementary file 1 — Table S1: Multivariate linear regression model for association between maternal depressive scores (by decile) and child growth Table S2: Multivariate linear regression model for association between maternal depressive scores (by decile) and child development Table S3: Multivariate logit regression for association between maternal depression and child undernutrition Table S4: Multivariate logit regression model for association between maternal depression and child development [file MCN-14-e12621-s001.docx]

**Supplemental Table 1: Multivariate linear regression model for association between maternal depressive scores (by decile) and child growth**

|  | **HAZ** | **WAZ** | **WHZ** | **Stunting** | **Underweight** | **Wasting** |
| --- | --- | --- | --- | --- | --- | --- |
|  | β | Β | β | OR | OR | OR |
| Decile 1 | 0 | 0 | 0 | 1 | 1 | 1 |
| Decile 2 | -0.03 | -0.02 | -0.04 | 1.01 | 0.93 | 1.03 |
| Decile 3 | 0.12 | 0.07 | -0.01 | 0.89 | 0.91 | 1.01 |
| Decile 4 | 0 | -0.07 | -0.09 | 0.99 | 1.07 | 1.28 |
| Decile 5 | 0.08 | 0.05 | 0.01 | 0.89 | 0.75 | 0.93 |
| Decile 6 | 0.11 | 0.07 | 0.01 | 0.88 | 0.74+ | 0.75 |
| Decile 7 | -0.09 | 0.05 | 0.14 | 1.1 | 0.84 | 1.05 |
| Decile 8 | 0.11 | 0 | -0.09 | 0.77 | 0.88 | 0.98 |
| Decile 9 | 0.07 | 0.02 | -0.07 | 1.01 | 0.94 | 1.15 |
| Decile 10 | -0.22* | -0.25** | -0.21* | 1.66* | 1.38 | 1.11 |

*p<0.05, **p<0.01, ***p<0.001

All models adjust for child age, gender, tribal caste, mother as HH head, number of children < 5, number of working age members, mother’s age, mother’s education, household SES. All models adjust for observational dependency across observations at the AWC catchment level.

**Supplemental Table 2: Multivariate linear regression model for association between maternal depressive scores (by decile) and child development**

|  | **Child development scores** | | | | | **Child development delay** | | | | |
| --- | --- | --- | --- | --- | --- | --- | --- | --- | --- | --- |
|  | **Communication** | **Gross motor** | **Fine motor** | **Problem solving** | **Personal social** | **Communication** | **Gross motor** | **Fine motor** | **Problem solving** | **Personal social** |
|  | β | β | Β | β | β | OR | OR | OR | OR | OR |
| Decile 1 | 1 | 1 | 1 | 1 | 1 | 1 | 1 | 1 | 1 | 1 |
| Decile 2 | -2.74** | -1.46 | -1.21 | 0.17 | -0.92 | 1.83** | 1.16 | 0.98 | 1.25 | 1.11 |
| Decile 3 | -1.62 | 0.17 | 0.14 | 1.09 | 0.66 | 1.47 | 0.71 | 1.09 | 1.05 | 0.81 |
| Decile 4 | -3.34** | -1.95 | -2.83* | -0.09 | -2.08+ | 1.60* | 1.04 | 1.34 | 1.19 | 1.07 |
| Decile 5 | -2.78* | -0.89 | -2.33+ | -0.25 | -0.74 | 1.74* | 1.04 | 1.25 | 1.3 | 1.05 |
| Decile 6 | -4.05** | -1.17 | -3.71** | -0.82 | -2.68* | 2.08** | 1.04 | 1.48+ | 1.19 | 1.31 |
| Decile 7 | -4.31** | -3.37** | -4.09* | -0.89 | -3.94** | 2.26*** | 1.65* | 1.67* | 1.13 | 1.63* |
| Decile 8 | -0.21 | -0.63 | -1.86 | -1.19 | 0.54 | 1.47 | 0.9 | 1.41 | 1.23 | 0.87 |
| Decile 9 | -0.29 | 0.14 | -2.56+ | 0.87 | -0.54 | 1.34 | 0.9 | 1.39 | 0.98 | 1.06 |
| Decile 10 | -0.92 | -0.98 | -4.85** | -1.46 | -1.58 | 1.74* | 1.04 | 1.39 | 1.50* | 1.17 |

*p<0.05, **p<0.01, ***p<0.001

All models adjust for child age, gender, tribal caste, mother as HH head, number of children < 5, number of working age members, mother’s age, mother’s education, household SES. All models adjust for observational dependency across observations at the AWC catchment level.

**Supplemental Table 3: Multivariate logit regression for association between maternal depression and child undernutrition**

|  | **Stunting** | | **Underweight** | | **Wasting** | |
| --- | --- | --- | --- | --- | --- | --- |
|  | Model 1 | Model 2 | Model 1 | Model 2 | Model 1 | Model 2 |
|  | OR | OR | OR | OR | OR | OR |
| Maternal depressive scores |  |  |  |  |  |  |
| Low depressive scores (<4) | Ref. | Ref. | Ref. | Ref. | Ref. | Ref. |
| High depressive scores (≥4) | 1.25* | 1.04 | 1.15 | 0.96 | 1.1 | 1.01 |
|  | **HAZ** | | **WAZ** | | **WHZ** | |
|  | Model 1 | Model 2 | Model 1 | Model 2 | Model 1 | Model 2 |
|  | Β | β | β | β | β | β |
| Maternal depressive scores |  |  |  |  |  |  |
| Low depressive scores (<4) | Ref. | Ref. | Ref. | Ref. | Ref. | Ref. |
| High depressive scores (≥4) | -0.11+ | 0.02 | -0.09+ | 0.02 | -0.03 | 0.02 |

+ p<0.10, *p<0.05, **p<0.01, ***p<0.001

Model 1 adjusts only for child age and gender. Model 2 adjusts for child age, gender, tribal caste, mother as HH head, number of children < 5, number of working age members, mother’s age, mother’s education and household SES. All models adjust for observational dependency across observations at the AWC catchment level.

**Supplemental Table 4: Multivariate logit regression model for association between maternal depression and child development**

|  | **Developmental delay** | | | | | | | | | | |
| --- | --- | --- | --- | --- | --- | --- | --- | --- | --- | --- | --- |
|  | **Communication** | | **Gross motor** | | **Fine motor** | | **Problem solving** | | **Personal social** | | |
|  | Model 1 | Model 2 | Model 1 | Model 2 | Model 1 | Model 2 | Model 1 | Model 2 | Model 1 | Model 2 | |
|  | OR | OR | OR | OR | OR | OR | OR | OR | OR | OR | |
| Maternal depressive scores |  |  |  |  |  |  |  |  |  |  | |
| Low depressive scores (<4) | Ref. | Ref. | Ref. | Ref. | Ref. | Ref. | Ref. | Ref. | Ref. | Ref. | |
| High depressive scores (≥4) | 2.02*** | 1.79*** | 1.21 | 1.06 | 1.63*** | 1.45** | 1.35* | 1.18 | 1.46*** | 1.31** | |
|  | **Developmental scores** | | | | | | | | | | |
|  | **Communication** | | **Gross motor** | | **Fine motor** | | **Problem solving** | | **Personal social** | | |
|  | Model 1 | Model 2 | Model 1 | Model 2 | Model 1 | Model 2 | Model 1 | Model 2 | Model 1 | | Model 2 |
|  | β | Β | β | β | β | β | β | β | β | | β |
| Maternal depressive scores |  |  |  |  |  |  |  |  |  | |  |
| Low depressive scores (<4) | Ref. | Ref. | Ref. | Ref. | Ref. | Ref. | Ref. | Ref. | Ref. | | Ref. |
| High depressive scores (≥4) | -4.87*** | -3.57*** | -2.02** | -1.10+ | -4.79*** | -3.18*** | -2.60*** | -1.32* | -2.34*** | | -1.29* |

+ p<0.10, *p<0.05, **p<0.01, ***p<0.001. Model 1 adjusts only for child age and gender. Model 2 adjusts for child age, gender, tribal caste, mother as HH head, number of children < 5, number of working age members, mother’s age, mother’s education and household SES. All models adjust for observational dependency across observations at the AWC catchment level.
